# Improving Trainee Onboarding Experience in a Child and Adolescent Psychiatry Higher Training Scheme in London: A Quality Improvement Project

**DOI:** 10.1192/bjo.2026.11494

**Published:** 2026-06-30

**Authors:** Anujavahinie Suntharamoorthy, Praveen Pandey

**Affiliations:** 1 East London NHS Foundation Trust (ELFT), London, United Kingdom; 2 North East London NHS Foundation Trust (NELFT), London, United Kingdom

## Abstract

**Aims:**

The RCPsych Recruitment and Retention Charter identifies good quality inductions and creating a culture of belonging as strategies to improve workforce wellbeing.

The North Central and East London Child and Adolescent Psychiatry Higher Training (NEL CAP HT) scheme incorporates 19 placements across four different trusts. After thepandemic, inconsistent one-hour online inductions were provided by current trainees for new starters without formal protected time.

To address uncertainty towards placements, anxieties when starting on-call work and to prevent risk to patient safety, a quality improvement project was undertaken:

- To improve NEL CAP trainee onboarding experienceby developing a standardised, mandatory, in-person induction.
- To improve morale, build trustand cohesiveness amongst trainees and trainers.

**Methods:**

Cycle 1 (August 2025) evaluated transition from fragmented online inductions to a structured half day, in-person session. Surveys measuring trainee confidence in onboarding andon-call experiences were completed by both previous trainees who had experience of online inductions and the new starters (n= 15).

Cycle 2 (January 2025) incorporated feedback from Cycle 1 to plan a full day in-person induction involving senior trainees and trainers, enriched with simulation case scenarios and a Microsoft Teams repository of relevant policies and protocols.

**Results:**

Quantitative data:

- Confidence in managing out of hours on-calls increased by 16%.
- Confidence in understanding the scheme structure and individual placements increased by 15%.

Qualitative themes:

Moving from an online to in-person induction led to a shift from trainees feeling isolated and anxious at the start of joining the scheme, to a sense of connectedness and reduced on-call anxieties.

Trainees frequently cited peer interaction and presence of trainers in the in-person induction as the most helpful part of their onboarding experience.

**Conclusion:**

In-person induction improves the scheme onboarding experience for trainees by reducing their anxieties and ensures patient safety. Trainer presence in higher trainee induction boosts morale and improves psychological safety for new starters. A formal full day induction incorporating timely changes to the scheme and on-call workflows using simulation-based learning, is expected to further enrich content delivery.

